# Plasma Neurofilament Light Chain: A Potential Biomarker for Neurological Dysfunction in Myalgic Encephalomyelitis/Chronic Fatigue Syndrome

**DOI:** 10.3390/biomedicines12071539

**Published:** 2024-07-11

**Authors:** Naiara Azcue, Beatriz Tijero-Merino, Marian Acera, Raquel Pérez-Garay, Tamara Fernández-Valle, Naia Ayo-Mentxakatorre, Marta Ruiz-López, Jose Vicente Lafuente, Juan Carlos Gómez Esteban, Rocio Del Pino

**Affiliations:** 1 Neurodegenerative Diseases Group, Biobizkaia Health Research Institute, 48903 Barakaldo, Spain; azkue.naiara@gmail.com (N.A.); beatriz.tijeromerino@osakidetza.eus (B.T.-M.); marianaceragil@gmail.com (M.A.); tamara.fernandezvalle@osakidetza.eus (T.F.-V.); naiayo@hotmail.com (N.A.-M.); marta.ruizlopez2@osakidetza.eus (M.R.-L.); 2 Department of Neurology, Cruces University Hospital-OSAKIDETZA, 48903 Barakaldo, Spain; 3 CIBERNED-CIBER, Institute Carlos III, 28029 Madrid, Spain; 4 Clinical Analysis Service, Cruces University Hospital, 48903 Barakaldo, Spain; raquel.perezgaray@osakidetza.eus; 5 Department of Neurosciences, University of the Basque Country UPV/EHU, 48940 Leioa, Spain; josevicente.lafuente@ehu.eus

**Keywords:** autonomic nervous system, cognition, myalgic encephalomyelitis/chronic fatigue syndrome, neurofilament light chain

## Abstract

Myalgic encephalomyelitis/chronic fatigue syndrome (ME/CFS) is a complex disorder characterized by heterogeneous symptoms, which lack specific biomarkers for its diagnosis. This study aimed to investigate plasma neurofilament light chain (NfL) levels as a potential biomarker for ME/CFS and explore associations with cognitive, autonomic, and neuropathic symptoms. Here, 67 ME/CFS patients and 43 healthy controls (HCs) underwent comprehensive assessments, including neuropsychological evaluation, autonomic nervous system (ANS) testing, and plasma NfL level analysis. ME/CFS patients exhibited significantly higher plasma NfL levels compared to HC (F = 4.30, *p* < 0.05). Correlations were observed between NfL levels and cognitive impairment, particularly in visuospatial perception (r = −0.42; *p* ≤ 0.001), verbal memory (r = −0.35, *p* ≤ 0.005), and visual memory (r = −0.26; *p* < 0.05) in ME/CFS. Additionally, higher NfL levels were associated with worsened autonomic dysfunction in these patients, specifically in parasympathetic function (F = 9.48, *p* ≤ 0.003). In ME/CFS patients, NfL levels explained up to 17.2% of the results in cognitive tests. Unlike ME/CFS, in HC, NfL levels did not predict cognitive performance. Elevated plasma NfL levels in ME/CFS patients reflect neuroaxonal damage, contributing to cognitive dysfunction and autonomic impairment. These findings support the potential role of NfL as a biomarker for neurological dysfunction in ME/CFS. Further research is warranted to elucidate underlying mechanisms and clinical implications.

## 1. Introduction

Myalgic encephalomyelitis/chronic fatigue syndrome (ME/CFS) is a complex syndrome characterized by heterogeneous symptoms, with a prominent emphasis on excessive fatigue. Common manifestations include post-exertional malaise, muscle pain, unrefreshing sleep, autonomic symptoms, cognitive dysfunction, and alterations in neuroendocrine and immune functions among affected patients [1,2,3,4]. At present, specific biomarkers supporting the diagnosis are lacking [5], necessitating reliance on a set of clinical criteria. Predominantly employed diagnostic criteria include those proposed by Fukuda et al. [6], the Canadian Consensus Criteria [7], and the International Consensus Criteria [4,8]. All these criteria converge on disabling fatigue, which is persistent and disproportionate to the effort exerted, as the primary symptom. The ICD-11 also defines it as post-viral fatigue syndrome (code 8E49), classifying it within nervous system disorders.

Although not all of the aforementioned diagnostic criteria require the syndrome to occur after a viral infection, it is considered that in most cases, fatigue appears following an infection by a virus or bacteria. Cases of ME/CFS have been described after infections by the Epstein–Barr virus, cytomegalovirus, and other pathogens [9]. Despite knowing the onset of the disease, the pathophysiology and underlying mechanisms responsible for the onset and establishment of the syndrome remain unknown.

Numerous studies have sought to comprehend the nature of the disease by investigating various genetic, immunological, metabolomic, endovascular, neurological, and physical dysfunction biomarkers, while specific biomarkers still remain elusive for ME/CFS [5]. Notably, altered natural killer cell profiles and elevated cytokine levels have been observed in ME/CFS compared to healthy controls (HC), indicating significant immunological dysregulation in these patients [10]. Furthermore, heightened titers of autoantibodies against muscarinic and adrenergic receptors have been identified [11], and differential factors have been pinpointed for up to eight genes involved in immunological functions [12]. Similarly, the stress mediator neuropeptide Y exhibited elevated levels in comparison to HC [13].

Recent studies highlighted the resemblance between post-COVID conditions and ME/CFS, suggesting a potential shared etiology for both syndromes [1,2,14,15,16]. Some studies have revealed increased plasma neurofilament light chain (NfL) levels in post-COVID patients, particularly in those with neurocognitive symptoms [17]. NfL, a protein component exclusive to the neurons’ cytoskeleton, is released into extracellular fluids during axonal damage, a predominant feature of numerous neurodegenerative processes leading to irreversible impairment [18]. NfL has been extensively studied in dementias such as Alzheimer’s disease [19] and frontotemporal dementia [20], as well as in other neurodegenerative diseases like amyotrophic lateral sclerosis [20] and multiple sclerosis [21]. Despite ongoing research, normative values for NfL concerning age and sex remain insufficient. Although studies primarily focus on analyzing NfL in cerebrospinal fluid (CSF) due to its greater specificity and sensitivity in establishing cutoff points, the concentration of NfL in plasma also presents advantages, being easy to obtain and demonstrating a moderate to high correlation with CSF NfL depending on the disease [18,22,23]. Investigating NfL in ME/CFS could provide valuable insights into the neurological implications of this disease. Notwithstanding these recent advancements, the underlying intricacies of this disease remain incompletely elucidated.

Therefore, this study aimed to compare plasma NfL values between ME/CFS patients and HC, exploring the potential of NfL as a biomarker for this pathology. Additionally, the study sought to examine the associations between plasma NfL levels and various clinical parameters, including cognition, fatigue, sleep quality, as well as autonomic and neuropathic signs, providing insights into the multifaceted manifestations of ME/CFS.

## 2. Materials and Methods

### 2.1. Participants and Demographic Data

We recruited 67 patients with ME/CFS through the Neurology Department at the Cruces University Hospital and 43 HC. Sex, disease duration, and neuropathic, autonomic, and neuropsychiatric symptoms were recorded for all participants.

General inclusion criteria included participants between 18 and 85 years old, with sufficient understanding and communication skills, who agreed to participate in the study. Patients diagnosed with CFS/ME should be previously diagnosed or meet the Fukuda et al. [6] criteria at the evaluation time. These criteria require the patient to have elevated fatigue for a minimum of 6 months, which cannot be explained by the physical or mental effort. The fatigue must be accompanied by four of the following symptoms: impaired memory, post-exertion malaise, unrefreshing sleep, arthralgia, headache, sore throat, and/or tender lymph nodes [6].

All patients with any metabolic or autoimmune diseases that could cause small fiber neuropathy (SFN) were discarded. Participants with pregnancy and/or lactation, severe trauma, alcoholism, drug addiction, severe heart disease, radiological diagnosis of brain structural pathology, concomitant diseases that could influence the results, as well as patients who have received some immunomodulatory treatments, were excluded.

### 2.2. Laboratory Procedures

Blood samples were collected in EDTA tubes for plasma NfL analysis. The samples were processed by centrifugation at 20 °C at 3599 rpm for 15 min to separate the plasma. Plasma was frozen in aliquots at −40 °C. All participants’ blood samples were taken and processed within 1 h. The samples were stored for three to six months. The determination of NfL was carried out on the LUMIPULSE^®^ G600II (Fujirebio, Malvern, PA, USA) analyzer [chemiluminescence (CLEIA)]. The automated method has a measurement range 2–500 pg/mL, with a limit of detection (LOD) 2.99 pg/mL, an analytical sensitivity (LOQ) of 3.25 pg/mL, and analytical imprecision of 3.1–4.3%. The intra-assay coefficient of variation is 2.6 to 4.1% vs. inter-assay, which is 0.0 to 3.1%. Automated methods, compared to classic ones such as ELISA, do not require duplicate samples, which is why they were not performed. Values of >20 pg/mL were taken as pathological, and 15–20 pg/mL as values of possible pathology [24,25].

### 2.3. Neuropsychologic and Neuropsychiatric Assessment

A comprehensive neuropsychological assessment was conducted, encompassing various domains. General cognition screening was evaluated using the Montreal Cognitive Assessment (MoCA). Attentional functions, including verbal and working memory, were assessed using Digits from the Wechsler Adult Intelligence Scale IV (WAIS-IV). Visual attention was measured using the Trail Making Test A (TMT A), sustained attention with the Touluose-Piéron Revised (TP-R), and alternating attention with the Trail Making Test B (TMT B). Verbal fluency was tested through categories such as animals and words beginning with the letter P. Processing speed was assessed using the Symbol Digit Modality Test (SDMT) and the Salthouse Perceptual Comparison Test (SPCT). Cognitive flexibility was evaluated using the Modified Wisconsin Card Sorting Test (M-WCST). Verbal memory was examined with the Hopkins Verbal Learning Test-Revised (HVLT-R), while visual memory was assessed using the Brief Visuospatial Memory Test-Revised (BVMT-R). Visuoconstructive capacity was tested with the Taylor Complex Figure Test (TCF) and visual perception with the Benton Judgment of Line Orientation (JLO). Inhibitory capacity and abstraction were assessed using the Stroop Test and similarities from WAIS-IV, respectively.

Neuropsychiatric and clinical status were also evaluated using specific questionnaires. General health was measured with the 36-Item Short Form Health Survey (SF-36). Fatigue levels and impact were assessed using the Modified Fatigue Impact Scale (MFIS). Depressive symptoms were evaluated using the Short Form of the Geriatric Depression Scale (GDS), anxiety symptoms with the State-Trait Anxiety Inventory (STAI), and suicidal ideation with the Columbia Suicide Severity Rating Scale (C-SSRS). Sleep quality was assessed using the Pittsburgh Sleep Quality Index (PSQI).

This comprehensive assessment provided a thorough understanding of the neuropsychological and clinical status of the participants, highlighting significant differences and areas of concern in patients with ME/CFS compared to HC.

### 2.4. Peripheral Nervous System

Autonomic nervous system (ANS) symptoms were comprehensively assessed using the Composite Autonomic Symptom Score (COMPASS-31). To evaluate hemodynamic autonomic function non-invasively, a Task Force Monitor (CNSystems, Graz, Austria) was utilized. Throughout the assessment, continuous monitoring of heart rate (HR) and blood pressure (BP) variability provided insights into autonomic regulation.

Parasympathetic and sympathetic functions were specifically evaluated using standardized techniques. Parasympathetic function was assessed through the deep breathing technique, where the expiration/inspiration (E/I) ratio and deep breathing index were derived from six successive cycles in the supine position. The Valsalva maneuver, performed at an expiratory pressure of 40 mmHg for 15 s, evaluated sympathetic function. The Valsalva index, calculated as the maximum HR divided by the lowest HR during the maneuver, provided further insights into autonomic parasympathetic responsiveness. The blood pressure recovery time (PRT), measuring the time for systolic BP (sBP) to return to baseline post-Valsalva maneuver, was also calculated.

During the 11 min Tilt Test at 60°, HR and BP values were recorded to assess autonomic function under orthostatic stress. All autonomic function tests were conducted under the supervision of experienced neurologists, ensuring accuracy and reliability in evaluating ANS dynamics in both patients with ME/CFS and HC.

Neuropathy assessment was focused on assessing SFN. SFN symptoms were evaluated using the Small Fiber Neuropathy Screening List (SFNSL). The functioning of nerve signals from ANS that controls sweat production was quantified non-invasively with the Sudoscan test (Impeto Medical, Paris, France) [26]. This device quantifies the electrochemical skin conductance (ESC) in palms and soles. Sensory SFN was assessed with the TSA-2 device (Medoc Advanced Medical Systems, Ramat Yishai, Israel), which includes contact heat and cold-evoked potentials and quantitative sensory testing (QST). For the CHEP (Contact Heat Evoked Potentials), 15 stimuli of 55 °C were performed every 30–45 s, and 15 stimuli of 9 °C every 30–45 s for the CEP (cold-evoked potentials). Latency (milliseconds, ms) and amplitude (microvolts, μV) values were obtained and analyzed.

### 2.5. Statistical Analysis

Statistical analyses were conducted using IBM SPSS Statistics for Windows, version 26.0 (IBM SPSS, Armonk, NY, USA). Assumptions of normality and homogeneity of variances for all variables were assessed prior to analysis.

Group differences in continuous variables were evaluated using Kruskal–Wallis tests, while categorical variables were assessed with Chi-square tests. Z values were calculated for all variables analyzed. ANCOVA was employed to examine differences between groups in neuropsychological, neuropsychiatric, autonomic, and neuropathic assessments, as well as plasma NfL levels. Age and sex were included as covariates in all analyses, and educational level was additionally considered for comparisons in neuropsychological and neuropsychiatric domains.

ROC curve analysis was performed to determine the utility of plasma NfL levels as a biomarker for distinguishing patients from HC. Pearson bivariate correlations were computed to explore relationships between plasma NfL levels and cognition, neuropsychiatric symptoms, clinical data, ANS function, and neuropathic signs.

Furthermore, stepwise linear regression was conducted to identify the variables that most significantly explained cognitive performance differences between patients and HC. Statistical significance was set at *p* < 0.05 (two-tailed) for all analyses. These comprehensive statistical methods provide robust insights into the associations and predictive value of plasma NfL levels across various domains in ME/CFS research.

## 3. Results

### 3.1. Participants

The demographic and clinical data are presented in Table 1. No statistically significant differences were observed between groups in terms of age. However, statistically significant differences were found in the educational level of participants, with the ME/CFS group exhibiting the lowest number of years in education. Additionally, significant distinctions emerged in clinical questionnaires, indicating that the patient group manifested a higher prevalence of autonomic and neuropathic symptoms, poorer general health, increased fatigue levels, diminished sleep quality, heightened anxiety–depressive symptoms, and suicidal ideation (Table 1).

### 3.2. Plasma NfL

**Figure 1:**
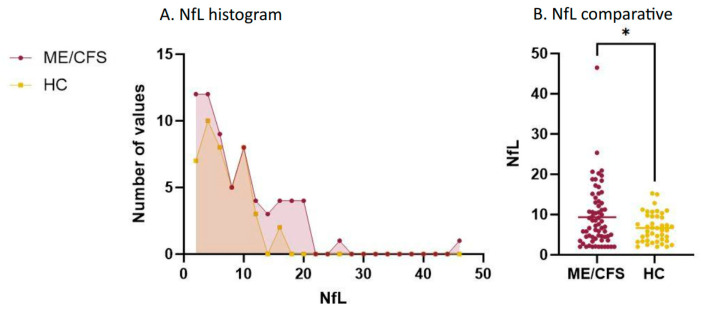
NfL levels per group. (**A**). NfL histogram. (**B**). NfL comparative. Note: NfL values are shown in pg/mL. * *p* < 0.050. HC: healthy controls; ME/CFS: myalgic encephalomyelitis/chronic fatigue syndrome; NfL: neurofilament light chain.

ME/CFS patients exhibited elevated plasma NfL levels compared to the HC group (F = 4.30, *p* = *0*.041), as depicted in Figure 1, even after adjusting for age and sex. The mean plasma NfL in patients was 8.80 ± 6.02 pg/mL, while in HC, the mean was 6.69 ± 3.57 pg/mL. Among the patients with ME/CFS, 13.43% had results considered possibly pathological (between 15–20 pg/mL), and 7.46% had pathological results (>20 pg/mL). In contrast, none of the HC presented pathological results, and only 4.65% had results, with none exceeding 16 pg/mL. However, despite the statistically significant variance in plasma NfL levels observed between the groups, the ROC curve analysis revealed that plasma NfL levels alone were insufficient for distinguishing patients from HC (AUC = 0.57, *p* < 0.05).

**Figure 2:**
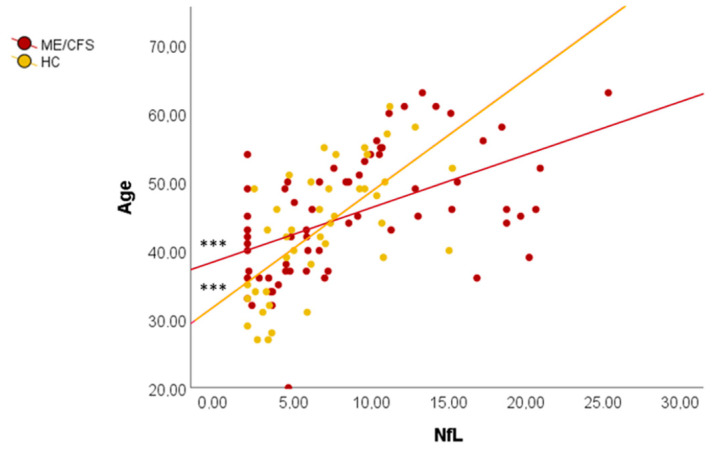
Linear regressions between NfL and age. *** *p* < 0.001. HC: healthy controls; ME/CFS: myalgic encephalomyelitis/chronic fatigue syndrome; NfL: neurofilament light chain.

The increase in plasma NfL levels was analyzed in relation to the age of the participants (Figure 2). Plasma NfL levels increased significantly the older the patients (F = 23.58, *p* = 0.000) and the HC (F = 28.97, *p* = 0.000). A greater increase in plasma NfL levels was observed in HC compared to patients, where the slope is more attenuated due to higher plasma NfL levels in younger individuals.

### 3.3. Neuropsychologic and Neuropsychiatric Results

The cognitive performance of both groups was meticulously analyzed and compared, taking into account the participants’ age, sex, and educational background. Patients diagnosed with ME/CFS demonstrated significantly poorer cognitive functioning across multiple domains when compared to the control group. Specifically, patients with ME/CFS showed deficits in general cognition (F = 23.22, *p* = 0.000), verbal fluency (F = 50.63, *p* = 0.000), processing speed (F = 43.56, *p* = 0.000), attention (F = 14.54, *p* = 0.000), verbal memory (F = 18.41, *p* = 0.000), visual memory (F = 7.15, *p* = 0.009), visuospatial perception (F = 7.09, *p* = 0.009), and abstraction (F = 22.39, *p* = 0.000).

In addition to cognitive deficits, the neuropsychiatric evaluation revealed substantial differences between the ME/CFS group and controls. Patients with ME/CFS reported significantly higher levels of physical and mental fatigue (F = 395.01, *p* = 0.000), poorer general health (F = 37.56, *p* = 0.000), and lower sleep quality (F = 73.20, *p* = 0.000). They also exhibited more severe depressive symptoms (F = 164.67, *p* = 0.000), anxiety symptoms (F = 50.47, *p* = 0.000), anxious personality traits (F = 6.91, *p* = 0.010), and suicidal ideation (F = 13.91, *p* = 0.000).

### 3.4. Peripheral Nervous System

The study of the ANS using the COMPASS-31 questionnaire revealed significantly greater autonomic symptoms in patients with ME/CFS compared to HC, even after adjusting for age and sex (F = 117.13, *p* = 0.000).

Further evaluation of ANS functioning identified notable differences between the groups. Cardiovascular function showed significant disparities (F = 4.04, *p* = 0.030), with patients with ME/CFS exhibiting abnormal HR responses both at rest (F = 14.70, *p* = 0.000) and during the tilt table test (F = 28.58, *p* = 0.000). Specifically, inappropriate tachycardia was observed in ME/CFS patients, meeting the diagnostic criteria for postural orthostatic tachycardia syndrome (POTS) in 31.3% of cases. POTS is characterized by a heart rate increase of more than 30 beats per minute or reaching at least 120 beats per minute upon standing, accompanied by symptoms such as dizziness, nausea, discomfort, or blurred vision.

Interestingly, no significant differences were found in tests assessing the responsiveness of the sympathetic and parasympathetic nervous systems, including the Valsalva maneuver and deep breathing tests, respectively.

The usual symptoms of SFN were also analyzed using the SFNSL, revealing significant differences between the two groups (F = 11.87, *p* = 0.001). In addition to the questionnaire, both objective and subjective quantitative tests were used to determine possible SFN. The results revealed that patients had worse heat detection than HC, demonstrated by higher latencies in CHEPs (F = 136.62, *p* = 0.000) and an increased threshold for perceiving heat as a noxious stimulus in QST (F = 6.92, *p* = 0.010).

### 3.5. Plasma NfL Implications

**Figure 3:**
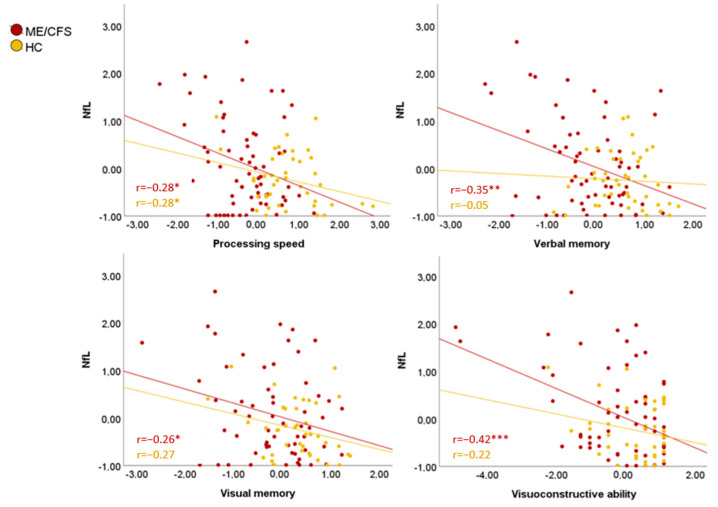
Plasma NfL correlations with cognition. * *p* < 0.05, ** *p* < 0.01, *** *p* ≤ 0.001. Note: values are shown in z-scores. ME/CFS: myalgic encephalomyelitis/chronic fatigue syndrome; NfL: neurofilament light chain.

Correlations of NfL levels and cognitive performance were analyzed, and results indicated a worse cognitive performance when higher plasma NfL levels in ME/CFS (Figure 3). Specifically, higher plasma NfL levels mainly indicated worse processing speed (r = −0.28, *p* = 0.025), both verbal memory (r = −0.35, *p* = 0.005) and visual memory (r = −0.26; *p* = 0.039), visuoconstructive ability (r = −0.42; *p* = 0.001), and lower total cognitive score (r = −0.38, *p* = 0.003). No statistically significant correlations were found between plasma NfL levels and cognitive performance in HC.

**Figure 4:**
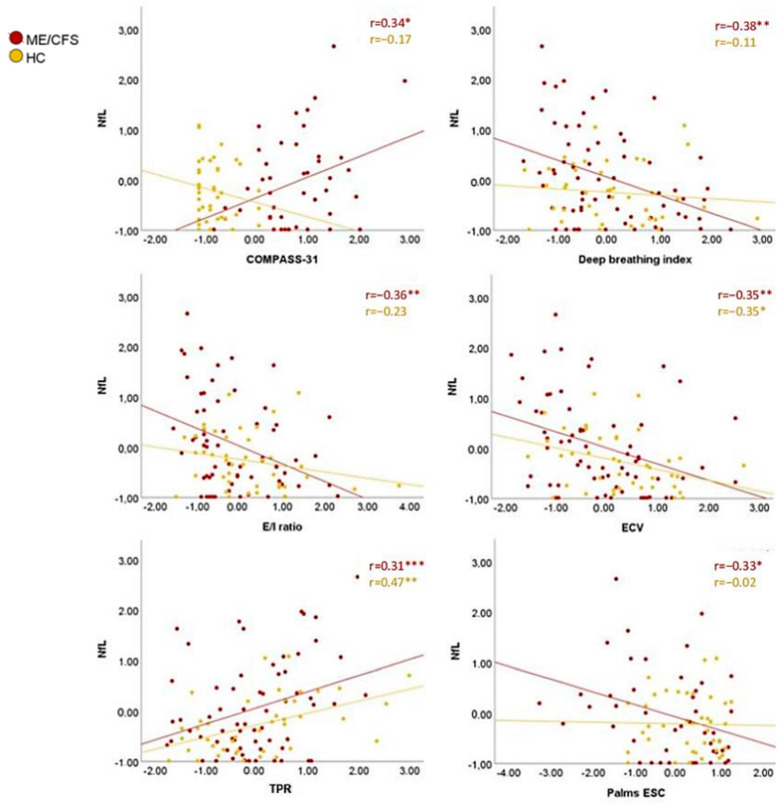
Plasma NfL correlations with ANS parameters. * *p* < 0.05, ** *p* < 0.01, *** *p* ≤ 0.001. Note: values are shown in z-scores. COMPASS-31: Composite Autonomic Symptom Score; ECV: extracellular cardiac volume; E/I ratio: expiration–inspiration ratio; ESC: electrochemical skin conductance; HC: healthy controls; ME/CFS: myalgic encephalomyelitis/chronic fatigue syndrome; NfL: neurofilament light chain; TPR: total peripheral resistance.

In addition to correlations with cognition, the results indicated higher plasma NfL levels in those with ANS impairment (Figure 4). A correlation was found between the COMPASS-31 autonomic symptoms questionnaire and NfL (r = 0.34; *p* = 0.023). Higher levels of NfL were related to worse parasympathetic function, both with the deep breathing index (r = −0.38; *p* = 0.002) and with the E/I ratio (r = −0.36, *p* = 0.002), indicating worse HR variability in the deep breathing technique, therefore, a higher NfL levels correlate with lower parasympathetic capacity. Higher levels of NfL were also associated with lower extracellular cardiac volume (ECV) (r = −0.35 *p* = 0.005) and higher total peripheral resistance (r = 0.31, *p* = 0.011). ESC in the palms of the hands was an indicator of functionality of the sympathetic cholinergic fibers of the ANS; higher levels of NfL were related to worse palm ESC (r = −0.33; *p* = 0.026) and also to cold detection in the QST (r = −0.36; *p* = 0.017).

In HC, higher levels of NfL are related to higher systolic (r = 0.37, *p* = 0.016) and diastolic blood pressure (r = 0.33, *p* = 0.032) in the supine position. As in patients, plasma NfL levels were also associated with lower ECV (r = −0.35, *p* = 0.023) and higher total peripheral resistance (r = 0.48, *p* = 0.002). No statistically significant correlations were found between NfL levels and neuropathy indicator variables.

The disparities between patients exhibiting pathological NfL values and those within the normal range were scrutinized. For this analysis, normal values of NfL were considered as 0–15 pg/mL, and values greater than 15 were considered pathological. This analysis was meticulously adjusted for both sex and age, uncovering statistically significant distinctions in key functional parameters of the parasympathetic nervous system, notably the deep breathing index (F = 8.24; *p* = 0.006) and the E/I ratio (F = 7.30; *p* = 0.009) in patients with ME/CFS. Additionally, variances in ECV were observed between patients with elevated and diminished plasma NfL levels (F = 4.45; *p* = 0.039), with those exhibiting higher NfL concentrations displaying diminished parasympathetic capacity and reduced ECV.

**Figure 5:**
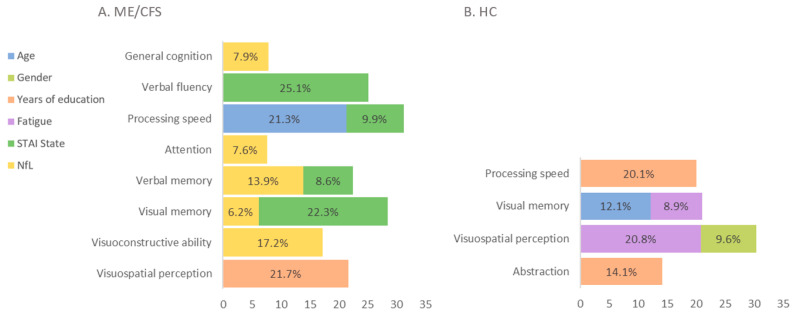
Linear regressions in cognition. (**A**). Stepwise linear regression in ME/CFS. (**B**). Stepwise linear regression in HC. HC: healthy controls; ME/CFS: myalgic encephalomyelitis/chronic fatigue syndrome; NfL: neurofilament light chain; STAI: State–Trait Anxiety Inventory.

Linear regressions adjusted for age and sex in ME/CFS patients revealed a relationship between plasma NfL and parasympathetic activation (F = 9.48, *p* = 0.003). Age, years of education, anxiety symptoms, and NfL levels were the variables that better explained cognition in ME/CFS. Plasma NfL levels did not have any implication in the cognitive performance of HC, unlike patients in whom NfL levels explained up to 17.2% of the results in cognitive tests (Figure 5).

## 4. Discussion

The objective of this study was to investigate the potential role of plasma NfL as a biomarker in ME/CFS and its relationship with autonomic, neuropathic, and cognitive symptoms in this pathology compared to HC.

Plasma NfL levels are indicative of axonal damage and have been extensively studied as a biomarker for degeneration within the central nervous system (CNS). Differences observed in these biomarker levels in our study suggested axonal harm in ME/CFS patients, a phenomenon, as seen in this sample, not solely attributable to aging. Elevated levels of NfL in plasma are observed in various conditions and pathologies, including neurodegenerative diseases, as well as acute conditions such as stroke, which do not necessarily lead to subsequent degenerative processes [19,20,23,27].

The chronic or recurrent viral infections frequently observed in many ME/CFS patients can trigger autoimmunity, consequently leading to inflammatory processes [28,29]. Additionally, there was evidence suggesting that ME/CFS patients exhibit a relative immunodeficiency, predisposing them to inadequate early infection control, resulting in chronic inflammatory responses to infectious insults [28,29,30,31]. Neurological and endocrine alterations described in ME/CFS patients support the notion of an inflammatory pathogenesis underlying the condition as a whole [32,33]. An inflammatory disease model also offers an explanation for the pronounced female sex bias associated with ME/CFS [34]. It was plausible to attribute some of the symptoms experienced by these patients to inflammatory processes, followed by subsequent neuronal damage and, therefore, higher levels of NfL [33]. It is noteworthy that case studies have revealed loss of cortical white matter and the presence of amyloid deposits [35]. Despite the elevated levels of NfL observed in these patients, current evidence does not support categorizing ME/CFS as a degenerative disease. This conclusion is drawn from the characteristic fluctuation of symptoms and the frequent occurrence of temporary remissions within this patient population [36]. Moreover, the syndrome’s overall severity often hinges on secondary factors such as anxiety, depression, and physical deconditioning. These factors contribute significantly to the variability and intensity of symptoms experienced by individuals with ME/CFS [1].

This study comprehensively assessed neuropsychological and neuropsychiatric manifestations in patients with ME/CFS compared to HC. The cognitive evaluation revealed significant deficits across multiple domains in ME/CFS, including general cognition, verbal fluency, processing speed, attention, verbal and visual memory, visuospatial perception, and abstraction. Concurrently, patients with ME/CFS reported markedly higher levels of physical and mental fatigue, poorer general health, and lower sleep quality. They also exhibited more severe depressive and anxiety symptoms, anxious personality traits, and higher rates of suicidal ideation.

Assessment of the ANS using the COMPASS-31 questionnaire underscored greater autonomic symptoms in ME/CFS, indicative of pervasive dysfunction in this cohort. Further examination revealed significant cardiovascular abnormalities, including inappropriate tachycardia during orthostatism, meeting the criteria for POTS in a substantial proportion of cases. Interestingly, no differences were found in tests assessing sympathetic and parasympathetic responsiveness.

Analysis of SFN symptoms indicated pronounced abnormalities in heat detection among ME/CFS patients, suggesting potential underlying neurophysiological impairments beyond cognitive and autonomic dysregulation. The extended latencies in the CHEPs and the worse detection of heat in the QST of these patients compared to the HC indicate a possible sensory neuropathy.

This study’s findings indicated a remarkable association between NfL levels and cognitive performance in ME/CFS patients. Unlike HC, cognitive performance in patients was significantly influenced by plasma NfL levels. Specifically, NfL levels accounted for up to 17.2% of cognitive performance variation, impacting general cognition, attention, verbal and visual memory, as well as visuconstructive capacity. ME/CFS has also been compared with post-COVID condition because of their similarities. Patients with severe COVID-19 sustained systemic inflammation had higher NfL concentrations, which predicted cognitive decline [37]. Future studies could perform periodic analyses on NfL levels and check whether this neural damage is related to inflammatory processes within the fluctuations inherent to the disease. The robust correlation observed between plasma NfL levels and cognitive symptoms supports the notion that axonal impairment predominantly manifests at the CNS level.

Plasma NfL as a biomarker of axonal damage in the CNS is well-established; recent efforts have focused on evaluating its utility as an indicator of peripheral axonal damage, particularly in neuropathies. Some studies suggest that this biomarker can effectively detect polyneuropathies in patients with hereditary transthyretin-related (ATTRv) amyloidosis [38] or chemotherapy-induced peripheral neuropathy (CIPN) [39]. However, conflicting findings have been reported in studies comparing patients with isolated fiber neuropathies to HC [40]. Thus, while the validity of plasma NfL in detecting peripheral neuropathies in these patients requires further validation, the variability in study outcomes underscores challenges in its application. Continued research efforts are essential to clarify the diagnostic utility of plasma NfL across different types of neuropathies. It is possible that NfL levels are useful in certain types of neuropathies but not in the small fiber one [40].

The correlations of NfL levels with parasympathetic and cognition functions give rise to the hypothesis that there is an affectation at the CNS, including areas involved in autonomic functions. One of the centers involved in the regulation of the ANS and respiratory control at a central level is the Locus Coeruleus (LC) [41]. The LC is a pontine nucleus that also mediates attention, memory, and arousal. Interestingly, attention and memory, along with visuoconstructive capacity, were the cognitive domains correlated with NfL levels in these patients. It is worth noting that several studies have found structural changes and/or hypoperfusion in the brainstem, so LC involvement is plausible [42]. The LC, by controlling arousal, has a strong implication in circadian rhythms, and this could also explain the insomnia of many ME/CFS patients. This nucleus also has implications for emotional responses, with decreased activity common in depression and dementia [43]. It would be interesting to analyze LC activity in these patients in future studies.

The strengths of this study included the sample size of ME/CFS patients and HC who underwent a thorough neuropsychologic and neuropsychiatric assessment, ANS evaluation, and NfL analysis. However, limitations included the lack of matching in years of education between the HC and patient groups, although statistical adjustments were applied to address this variable.

Future research should further explore the role of NfL in differentiating between neuropathic conditions and its implications for ME/CFS management. Despite its limitations, this study contributes valuable insights into the multifaceted nature of ME/CFS and underscores the potential utility of plasma NfL as a biomarker in understanding and managing this complex syndrome.

## 5. Conclusions

Plasma NfL levels were significantly higher in ME/CFS patients compared to HC. The study highlighted significant deficits in cognitive domains among ME/CFS patients, alongside higher levels of fatigue, poorer general health, and lower sleep quality. Interestingly, plasma NfL levels correlated with cognitive performance and parasympathetic function in ME/CFS, reinforcing the hypothesis of CNS involvement in ME/CFS pathogenesis. These findings underscore the potential role of plasma NfL as a biomarker for neurologic dysfunction in ME/CFS. However, further research is needed to elucidate the underlying mechanisms and clinical implications of these associations.

## Figures and Tables

**Table 1:** Sociodemographic and clinical data.

	ME/CFS (*n* = 67) M (SD)	HC (*n* = 43) M (SD)	Statistics
Age, years	44.63 (10.42)	43.19 (9.18)	U = 1260.00
Education, years	15.19 (5.52)	16.45 (2.72)	U = 1764.50 *
Female, n (%)	61 (91.00%)	34 (79.10%)	*χ*^2^ = 3.19
Disease duration, months	60.59 (59.07)	-	-
COMPASS-31	25.24 (10.79)	4.13 (4.73)	U = 73.00 ***
SFNSL	34.51 (15.86)	2.46 (3.68)	U = 34.50 ***
SF-36	30.48 (16.19)	85.81 (9.04)	U = 2653.00 ***
MFIS	66.04 (14.95)	10.19 (11.73)	U = 21.50 ***
PSQI	12.84 (4.78)	5.24 (3.13)	U = 283.00 ***
STAI-State	30.37 (15.49)	11.47 (4.37)	U = 424.50 ***
STAI-Trait	20.98 (15.64)	13.21 (8.79)	U = 1010.00 *
GDS	8.92 (3.72)	1.02 (1.45)	U = 53.00 ***
C-SSRS	1.00 (1.74)	0.00 (0.00)	U = 840.00 ***
**Cognitive performance (z-scores)**			
General cognition	−0.12 (0.25)	0.19 (0.07)	F = 22.83 ***
Verbal fluency	−0.27 (0.81)	2.48 (0.88)	F = 55.66 ***
Processing speed	−0.34 (0.76)	2.83 (0.73)	F = 48.43 ***
Attention	−0.34 (0.58)	0.73 (0.07)	F = 16.89 ***
Verbal memory	−0.14 (0.85)	0.57 (0.58)	F = 23.25 ***
Visual memory	−0.22 (0.84)	0.31 (0.63)	F = 12.15 ***
Visuoconstructive ability	−0.23 (1.26)	0.28 (0.84)	F = 5.07 *
Visuospatial perception	−0.25 (0.96)	0.36 (0.74)	F = 11.92 ***
Abstraction	−0.40 (0.87)	0.47 (0.78)	F = 27.76 ***
Executive functions	−0.41 (0.26)	−0.37 (0.38)	F = 0.28

* *p* ≤ 0.05; *** *p* ≤ 0.001. Note: These results are not adjusted for age, sex and education. COMPASS: The Composite Autonomic Symptom Score; C-SSRS: Columbia Suicide Severity Rating Scale; GDS: Geriatric Depression Scale; HC: healthy controls; ME/CFS: myalgic encephalomyelitis/chronic fatigue syndrome, MFIS: modified fatigue impact scale; PSQI: Pittsburgh Sleep Quality Index; SF-36: The 36-Item Short Form Health Survey; SFNSL: Small Fiber Neuropathy Screening List; STAI: State-Trait Anxiety Inventory.

## Data Availability

The data presented in this study are available on request from the corresponding author. The data are not publicly available due to privacy reasons.
